# Apoptosis gene expression analysis identifies biologically distinct AML clusters and *BIK* as an independent prognostic marker

**DOI:** 10.1007/s00277-026-07268-7

**Published:** 2026-09-10

**Authors:** Kinga Strzałka, Piotr Strzałka, Dariusz Jarych, Aneta Wiśnik, Damian Mikulski, Kacper Kościelny, Magdalena Czemerska, Izabela Zawlik, Agnieszka Wierzbowska, Agnieszka Pluta

**Affiliations:** 1 Department of Hematology, Multidisciplinary Provincial Center of Traumatology and Oncology Nicolas Copernicus in Lodz, Medical University of Lodz, Lodz, Poland; 2 Multidisciplinary Provincial Center of Traumatology and Oncology Nicolas Copernicus in Lodz, Lodz, Poland; 3 Laboratory of Virology, Institute of Medical Biology, Polish Academy of Sciences, Lodz, Poland; 4 Department of Biostatistics and Translational Medicine, Medical University of Lodz, Lodz, Poland; 5 Department of General Genetics, Faculty of Medicine, Collegium Medicum, University of Rzeszow, Rzeszow, Poland

**Keywords:** Acute myeloid leukemia, Apoptosis, Gene expression profiling, Venetoclax

## Abstract

Dysregulation of apoptosis is a key feature of acute myeloid leukemia (AML) and contributes to therapy resistance, yet the clinical relevance of apoptosis-related gene expression remains incompletely understood. We prospectively analyzed mRNA expression of apoptosis-related genes in bone marrow mononuclear cells from 90 newly diagnosed AML patients. Gene expression clustering identified two biologically distinct patient groups. The subgroup with significantly inferior overall survival (Cluster 2) showed coordinated upregulation of pro-apoptotic genes, including *BCL2L11 (BIM)*,* BBC3 (PUMA)*,* BID*,* BAD*,* BIK*, and *BAX*, together with a shift in anti-apoptotic signaling toward lower *BCL2* expression and increased expression of alternative survival regulators, *MCL1* and *BCL2L1 (BCL-xL)*. Reduced expression of executioner caspases *CASP3* and *CASP7* was also observed in Cluster 2, which may indicate less efficient apoptotic execution. Among patients treated with venetoclax + azacitidine (VenAza), Cluster 2 demonstrated significantly shorter event-free survival. This finding is consistent with the biological features of Cluster 2, including a monocytic immunophenotype previously associated with reduced sensitivity to BCL-2–targeted therapy and increased reliance on alternative anti-apoptotic regulators such as MCL1 and BCL-xL. Analysis of individual genes showed that higher *BIK* mRNA expression was associated with poorer survival and lack of response to VenAza, highlighting the paradoxical association between increased expression of this pro-apoptotic gene and adverse clinical outcome. These findings demonstrate that dysregulation of apoptosis-related gene expression defines clinically distinct AML subgroups and may contribute to differences in prognosis and therapeutic response.

**Supplementary Information:**

The online version contains supplementary material available at https://doi.org/10.1007/s00277-026-07268-7.

## Introduction

Acute myeloid leukemia (AML) is a clinically and molecularly heterogeneous disease characterized by uncontrolled proliferation of immature myeloid cells, leading to accumulation of abnormal blasts and suppression of normal hematopoiesis [1, 2]. Molecular instability is a hallmark of AML, with numerous pathogenic variants promoting oncogene activation and evasion of regulated cell death (RCD) [3].

RCD plays a central role in preventing oncogenesis, and its best-known form, apoptosis, is essential for maintaining tissue integrity, particularly in rapidly renewing tissues such as the hematopoietic system [3]. Apoptosis occurs through two major pathways: intrinsic (mitochondrial) and extrinsic (death receptor). The intrinsic pathway is triggered by cellular stress and involves mitochondrial outer membrane permeabilization (MOMP), regulated by BCL-2 family proteins. MOMP leads to the release of apoptogenic factors, activation of caspase 9 and executioner caspases (3, 6, and 7), and ultimately programmed cell death [4].

The BCL-2 protein family is a key regulator of intrinsic apoptosis and tightly controls MOMP. It consists of three functional groups: pro-apoptotic BH3-only members (BIM, BID, PUMA, NOXA, HRK, BMF, and BAD); pro-apoptotic effector proteins (BAX and BAK); and anti-apoptotic members (BCL-2, BCL-xL, BCL-W, MCL-1, BCL-2A1, and BCL-B) [2]. The balance among these proteins and their regulators determines cellular susceptibility to apoptosis [2].

Dysregulated expression of programmed cell death genes is common in hematologic malignancies, particularly AML, promoting the survival of genetically unstable cells and selection of aggressive clones [2]. Therapies targeting apoptotic pathways have improved treatment options for AML patients ineligible for intensive therapy. However, among BCL-2 family inhibitors, only venetoclax has received FDA and EMA approval. Resistance to venetoclax remains a major challenge and is frequently associated with increased expression of alternative anti-apoptotic proteins such as MCL-1 and BCL-xL [5, 6]. Therefore, therapeutic approaches targeting additional BCL-2 family members are being explored to overcome resistance to BCL-2 inhibition [6].

Comprehensive analyses of apoptosis-related gene expression in well-characterized AML cohorts remain limited. Therefore, this study aimed to assess the prognostic and clinical relevance of mRNA expression of selected apoptosis pathway genes in AML.

## Materials & methods

###  Study cohort

Bone marrow samples were prospectively collected from 98 consecutive patients with newly diagnosed AML between September 2021 and June 2024. After exclusion of eight samples with missing gene expression data, 90 patients were included in the final analysis. Treatment response was assessed according to ELN 2022 criteria [7]. Detailed treatment regimens and laboratory procedures are provided in the Supplementary Methods. The study was conducted in accordance with the Declaration of Helsinki and approved by the local ethics committee. All participants provided written informed consent.

### RNA isolation and gene expression analysis

Total RNA was isolated from bone marrow mononuclear cells and reverse transcribed into cDNA. Expression of *BCL2L11 (BIM)*,* BID*,* BIK*,* BBC3 (PUMA)*,* PMAIP1 (NOXA)*,* BAD*,* BAX*,* BAK1*,* BCL2*,* BCL2L1 (BCL-xL)*,* MCL1*,* SMAC/DIABLO*,* CASP3*,* CASP7*, and *TP53*^wt^ was quantified using TaqMan real-time PCR assays. Expression data were normalized to *MRPL19* and *PPIA*, selected as the most stable reference genes using NormiRazor software [8]. Relative expression was calculated using the ΔCt method and fold changes (FC) using the ΔΔCt method. A Ct value ≤ 37 was considered indicative of detectable gene expression. Detailed experimental procedures are provided in the Supplementary Methods.

### Statistical analysis

Survival was analyzed using Kaplan–Meier estimates and Cox proportional hazards regression models. Variables with clinical relevance and *p* < 0.1 in univariate analyses were included in multivariable models. Hierarchical clustering was performed using apoptosis-related gene expression profiles. Detailed statistical methods are provided in the Supplementary Methods.

## Results

### Patient characteristics and clinical predictors of outcome

A total of 90 patients were included. The median age at diagnosis was 66.0 years (IQR 55.2–73.0). According to ELN 2022 risk stratification, 48.9% of patients were classified as adverse risk, 35.6% as intermediate risk, and 12.2% as favorable risk. Treatment approaches were heterogeneous: 36.7% received intensive therapy, 27.8% venetoclax plus azacitidine (VenAza), and 35.6% other non-intensive treatments. Baseline clinical characteristics are summarized in Table 1.

**Table 1:** Baseline demographic, clinical, and laboratory characteristics of the study cohort

Variable	Whole cohort (*N* = 90)
Sex, female - n (%)	39 (43.3)
Age at diagnosis - median (IQR)	66.0 (55.2–73.0)
Age > 65 years - n (%)	52 (57.8)
ECOG - n (%)	
0–1	57 (63.4)
2	20 (22.2)
≥ 3	13 (14.4)
HCT-CI - n (%)	
0	28 (31.1)
1–2	33 (36.7)
3	17 (18.9)
≥ 4	12 (13.3)
Treatment - n (%)	
Intensive	33 (36.7)
VenAza	25 (27.8)
Non-intensive	32 (35.6)
Response according to treatment - n (%)	
Intensive chemotherapy (*n* = 33)	
CR	28 (84.9)
PR	1 (3.0)
NR	1 (3.0)
ED	3 (9.1)
VenAza ^§^ (*n* = 25)	
CR	13 (52.0)
PR	5 (20.0)
NR	4 (16.0)
ED	3 (12.0)
Other non-intensive treatment (*n* = 32)	
CR	1 (3.1)
PR	7 (21.9)
NR	14 (43.8)
ED	10 (31.2)
alloHSCT - n (%)	26 (28.9)
sAML - n (%)	25 (27.8)
Cytogenetic risk according to MRC - n (%)	
favorable	5 (5.6)
intermediate	58 (64.4)
adverse	27 (30.0)
Cytogenetic-molecular risk according to ELN 2022 - n (%)	
favorable	11 (12.2)
intermediate	32 (35.6)
adverse	44 (48.9)
unavailable	3 (3.3)
MDS-related cytogenetics - n (%)	34 (39.1)
Laboratory parameters at diagnosis	Median (IQR)
Blasts, PB (%)	17.0 (3.0–60.0)
Blasts, BM (%)	44.5 (24.0–68.0)
WBC (×10⁹/L)	15.0 (3.0–72.5)
ANC (×10⁹/L)	2.8 (0.6–5.8)
Hb (g/dL)	8.9 (7.7–9.9)
Plt (×10⁹/L)	55.0 (28.0–105.0)
CRP (mg/L)	19.0 (6.0–78.0)
Elevated CRP - n (%)	69 (76.7)
LDH (U/L)	401.0 (234.0–735.0)
Elevated LDH - n (%)	60 (66.7)
Uric acid (mg/dL)	5.8 (4.5–7.2)
Elevated uric acid - n (%)	38 (42.2)
Albumin (g/dL)	3.8 (3.4–4.1)
Low albumin - n (%)	31 (35.2)

^§^ Treatment response was assessed after the first cycle of VenAza, with re-evaluation after the second cycle in patients without evidence of response after first cycle

*ECOG* Eastern Cooperative Oncology Group performance status, *HCT-CI* Hematopoietic Cell Transplantation Comorbidity Index, *alloHSCT* allogeneic hematopoietic stem cell transplantation, *sAML* secondary acute myeloid leukemia, *ELN* European LeukemiaNet, *MRC* Medical Research Council, *CR* complete remission, *PR* partial remission, *NR* no response, *ED* early death, *VenAza* venetoclax plus azacitidine, *PB* peripheral blood, *BM* bone marrow, *WBC* white blood cell count, *ANC* absolute neutrophil count, *Hb* hemoglobin, *Plt* platelet count, *CRP* C-reactive protein, *LDH* lactate dehydrogenase, *MDS* myelodysplastic syndrome

**Fig. 1:**
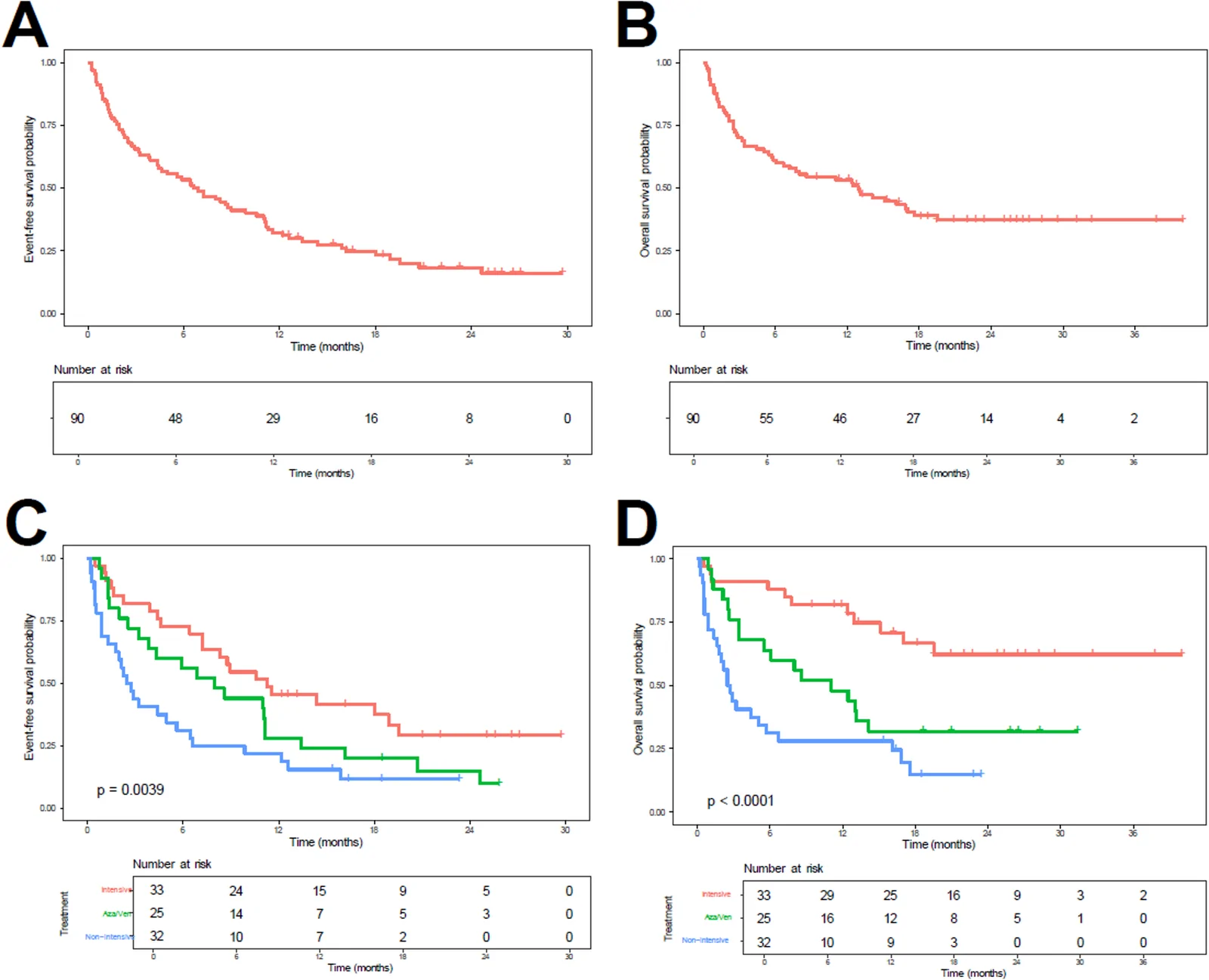
Kaplan–Meier curves for event-free survival (EFS) and overall survival (OS) in the entire cohort (A, B) and according to treatment intensity (C, D)

For the entire cohort, the median EFS was 6.7 months (95% CI 4.4–11.0), and the median OS was 12.9 months (95% CI 6.7–19.5) (Fig. 1A, B). In univariate Cox analysis, non-intensive treatment vs. intensive therapy (HR 2.55, 95% CI 1.45–4.51) was significantly associated with inferior EFS (Fig. 1C; Supplementary Table 1). Consistently, in the univariate OS analysis, treatment intensity showed a pronounced impact: compared with intensive therapy, non-intensive treatment was associated with markedly worse OS (HR 4.76, 95% CI 2.33–9.71; *p* < 0.0001), whereas VenAza therapy was associated with intermediate outcomes (HR 2.58, 95% CI 1.20–5.51; *p* = 0.0147) (Fig. 1D; Supplementary Table 2).

## Identification of apoptosis gene expression clusters

**Fig. 2:**
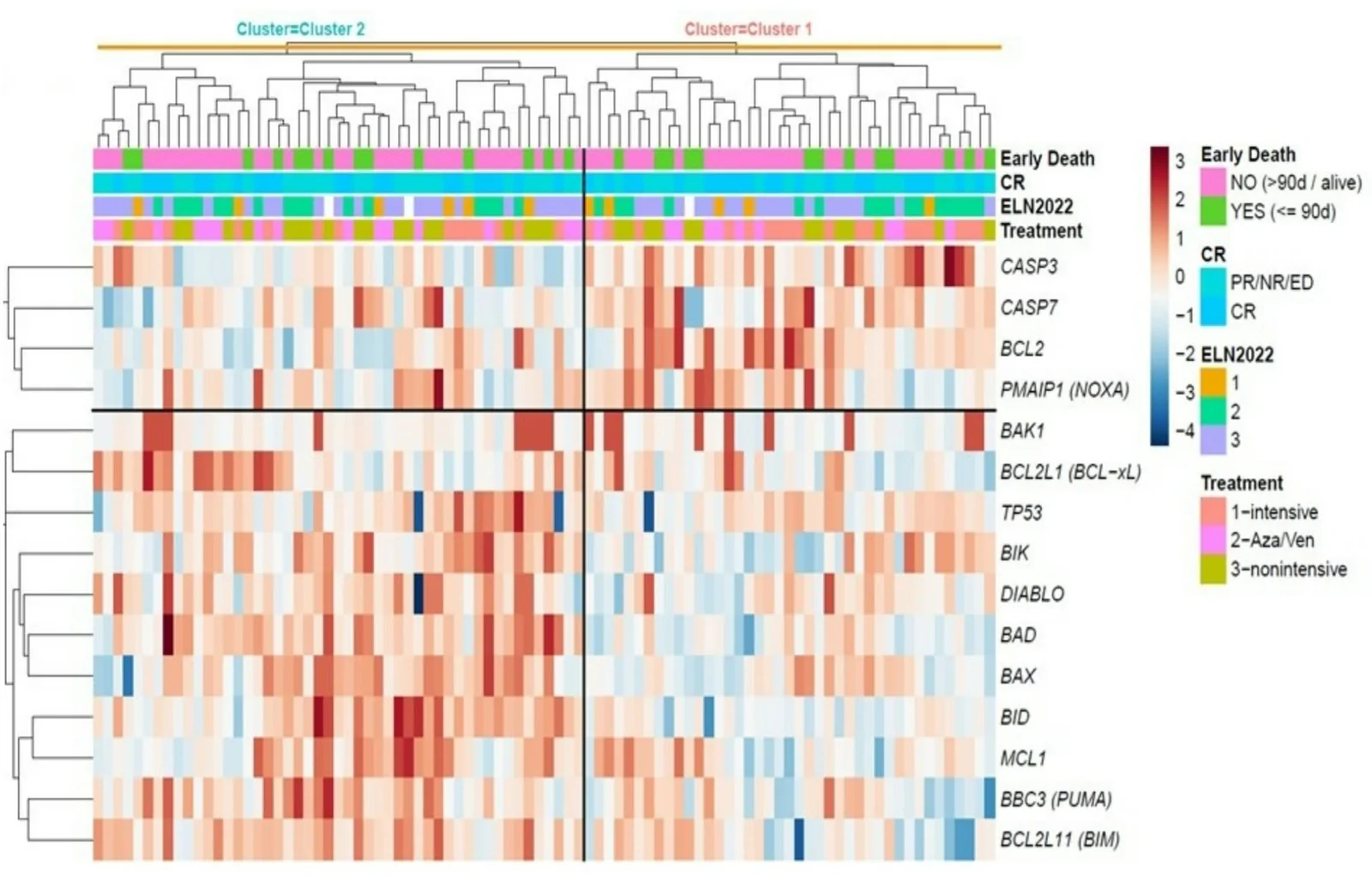
Unsupervised clustering of apoptosis-related gene expression identifies two apoptosis-related transcriptional subgroups in AML. Heatmap of z-score normalized ΔCt expression values of apoptosis-related genes in AML patients, clustered using Spearman correlation distance and complete linkage. Patients are ordered according to cluster assignment. Clinical annotations indicate ELN 2022 risk category, treatment regimen, complete remission status, and early death

Unsupervised hierarchical clustering based on apoptosis-related gene expression identified two distinct patient clusters (Fig. 2). Cluster 2 was characterized by coordinated upregulation of several pro-apoptotic genes, including *BAD* (FC 2.06), *BID* (FC 1.80), *BCL2L11/BIM* (FC 2.56), *BBC3/PUMA* (FC 1.99), *BIK* (FC 2.43), *BAX* (FC 1.28), together with increased expression of the anti-apoptotic genes *MCL1* (FC 1.40) and *BCL2L1 (BCL-xL)* (FC 1.72). This was accompanied by downregulation of *CASP3* (FC 0.52), *BCL2* (FC 0.54), and *CASP7* (FC 0.84) (Supplementary Table 3; Supplementary Fig. 1).

**Fig. 3:**
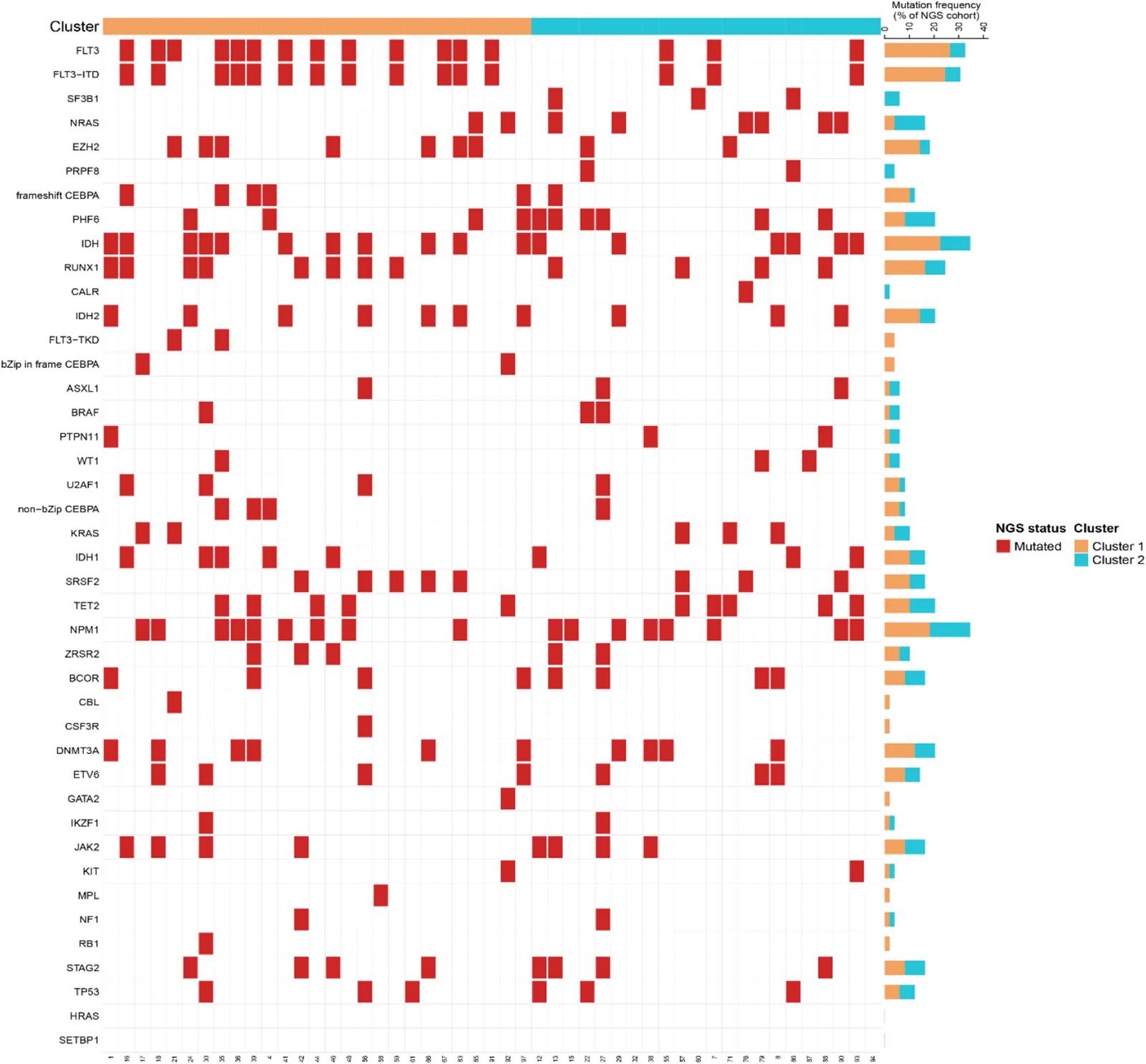
Genomic characteristics of apoptosis-defined AML clusters. Oncoplot showing recurrent somatic mutations in patients with available NGS data, ordered according to apoptosis cluster assignment and by increasing p-value, with the most significant associations shown at the top. Mutations are indicated in red and wild-type status in white. Stacked bars on the right show the frequency of each mutation in Cluster 1 and Cluster 2 for each recurrent molecular abnormality. Statistical comparisons of mutation frequencies between clusters are provided in Supplementary Table 4. Abbreviations: NGS, next-generation sequencing; WT, wild type; MUT, mutated.

**Fig. 4:**
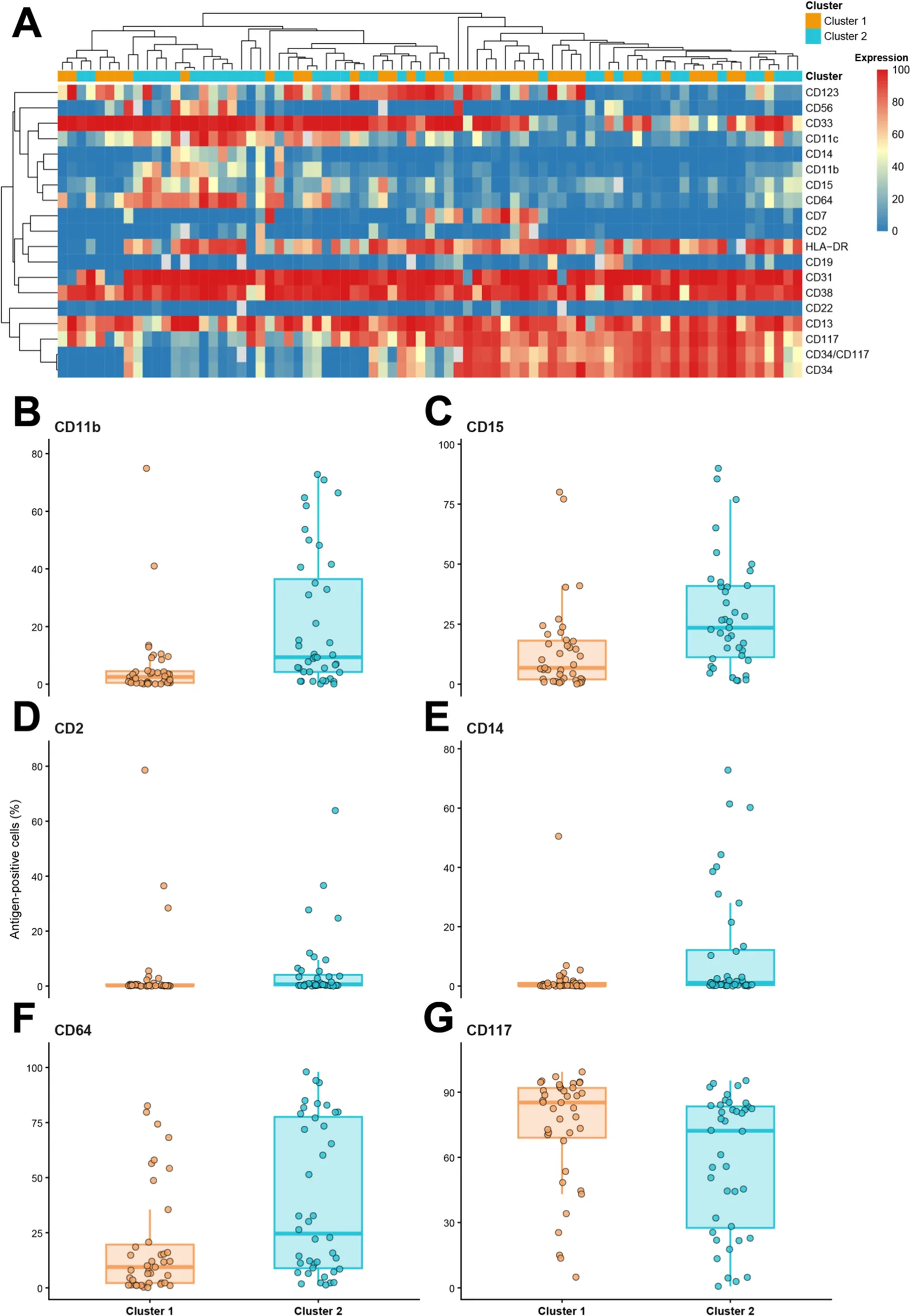
Immunophenotypic characteristics of apoptosis-defined AML clusters. (**A**) Heatmap showing immunophenotypic marker expression (percentage expression, 0–100%) across patients. Marker expression was scaled per marker, and patients were clustered using Spearman distance and complete linkage. Patients are annotated according to cluster assignment. (**B**–**G**) Dot–box plots showing individual patient values for the six immunophenotypic markers that differed significantly between clusters after false discovery rate (FDR) adjustment: CD11b (**B**), CD15 (**C**), CD2 (**D**), CD14 (**E**), CD64 (**F**), and CD117 (**G**). Box plots indicate the median and interquartile range (IQR)

With respect to the mutational landscape, Cluster 2 showed a significantly lower frequency of *FLT3* mutations compared with Cluster 1 (12.5% vs. 52.0%, *p* = 0.0050) (Fig. 3; Supplementary Table 4). Comparative analysis of immunophenotypic profiles revealed marked differences between clusters (Fig. 4A-G; Supplementary Table 5). Cluster 2 displayed significantly higher expression of myeloid- and monocytic-associated antigens, including CD11b (median 9.4 vs. 2.5; adjusted *p* = 0.0017), CD15 (23.5 vs. 6.8; adjusted *p* = 0.0036), CD14 (1.1 vs. 0.2; adjusted *p* = 0.0091), and CD64 (24.6 vs. 9.4; adjusted *p* = 0.0091). CD2 expression was also higher in Cluster 2 (0.9 vs. 0.2; adjusted *p* = 0.0091), whereas CD117 expression was significantly higher in Cluster 1 (85.2 vs. 72.0; adjusted *p* = 0.0190).

Baseline clinical characteristics according to cluster assignment are shown in Supplementary Table 6**.** The two clusters did not differ significantly with respect to age, sex, performance status, comorbidity burden, or other routine clinical parameters. In contrast, leukemic burden at diagnosis differed markedly. Patients in Cluster 1 had significantly higher peripheral blood blast count (median 53.0% vs. 9.0%, *p* = 0.0004) and higher bone marrow blast infiltration (median 54.0% vs. 37.0%, *p* = 0.0039) compared with Cluster 2.

**Fig. 5:**
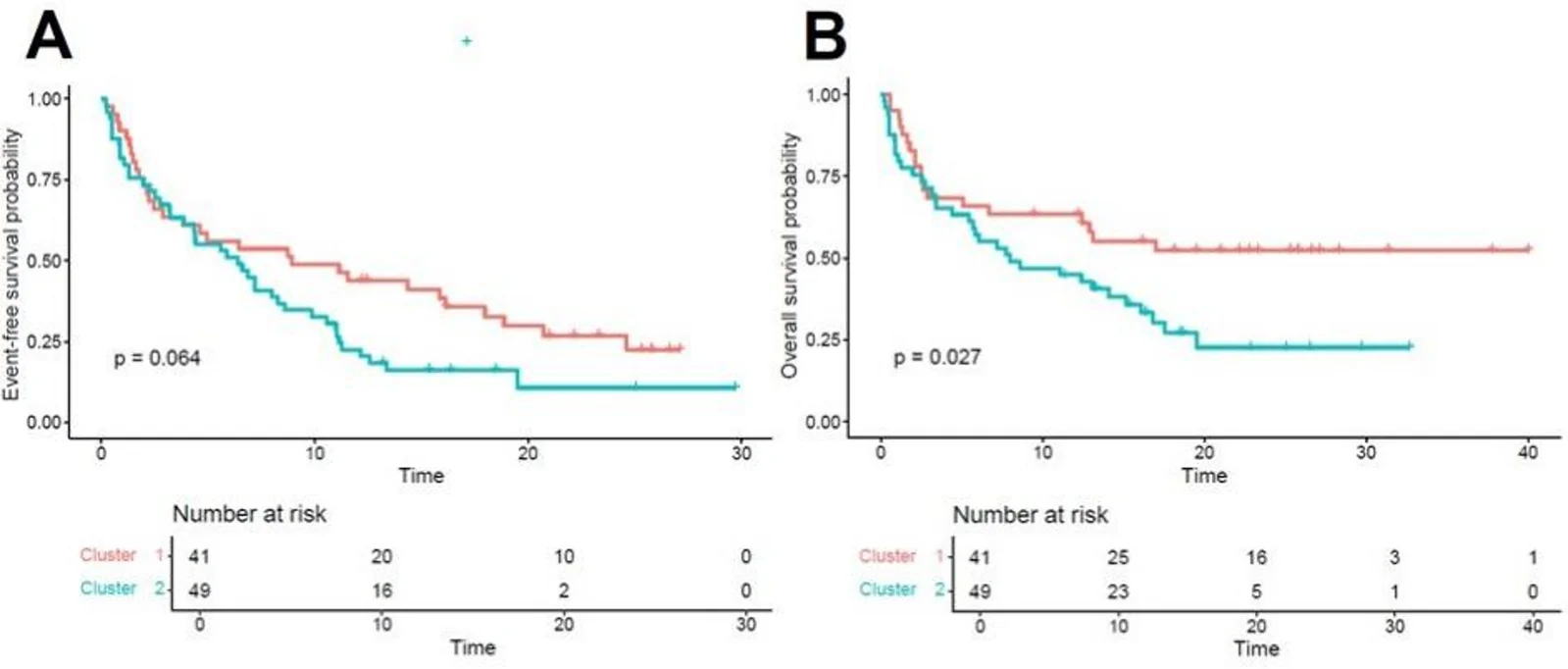
Clinical outcomes according to apoptosis-related gene expression clusters. (**A**) Kaplan–Meier curve for event-free survival (EFS) according to cluster membership. (**B**) Kaplan–Meier curve for overall survival (OS) demonstrating significantly worse survival in Cluster 2 (log-rank *p* = 0.027)

Cluster assignment was associated with clinical outcomes. Patients in Cluster 2 had inferior OS (log-rank *p* = 0.027) and showed a trend toward shorter EFS (*p* = 0.064) (Fig. 5A, B). In multivariable Cox analysis for EFS, Cluster 2 remained an independent adverse prognostic factor (HR 2.10, 95% CI 1.26–3.49, *p* = 0.0044), together with ECOG ≥ 2 (HR 4.33, 95% CI 2.47–7.58, *p* < 0.0001) (Supplementary Table 7). Similarly, in the OS model, Cluster 2 independently predicted inferior survival (HR 2.14, 95% CI 1.19–3.87, *p* = 0.0113), along with ECOG ≥ 2 (HR 4.00, 95% CI 2.14–7.51, *p* < 0.0001) (Supplementary Table 8).

## C. Patients treated with VenAza regimen

**Fig. 6:**
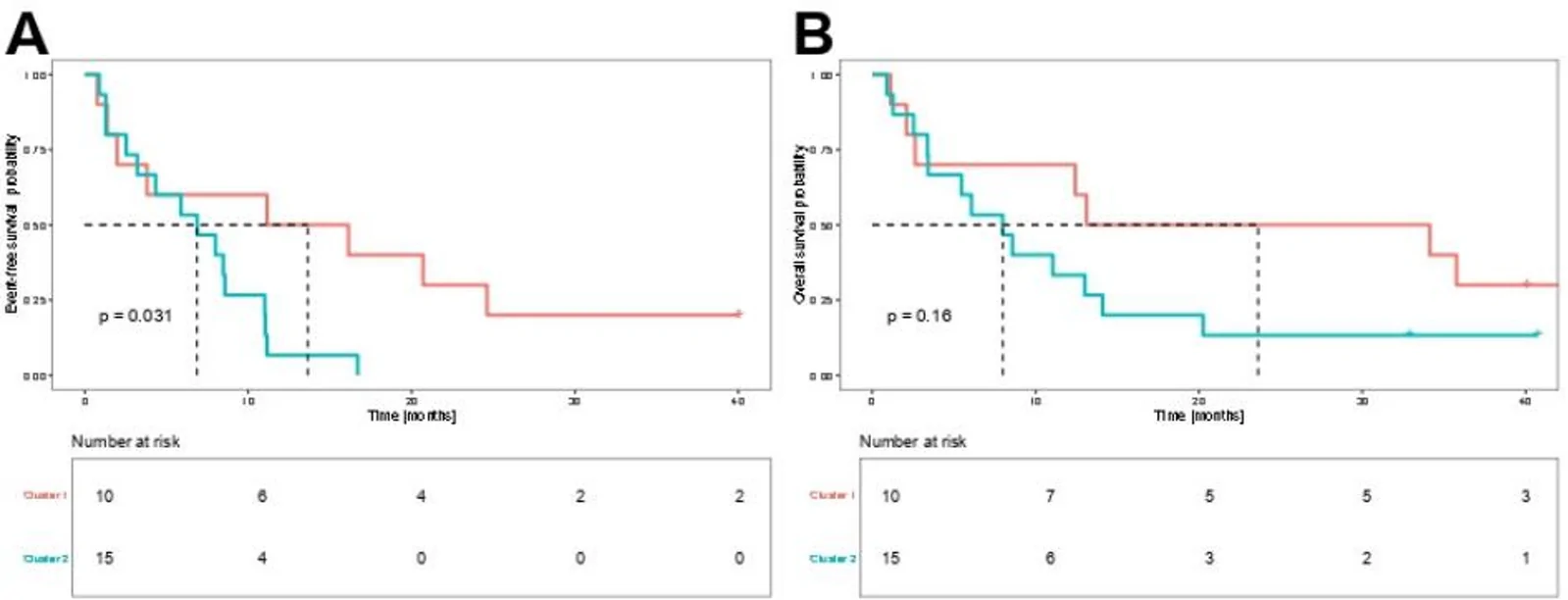
Kaplan–Meier survival analysis in patients treated with venetoclax + azacitidine (VenAza) according to cluster assignment. (**A**) Event-free survival (EFS), demonstrating significantly shorter EFS in Cluster 2 (log-rank *p* = 0.031). (**B**) Overall survival (OS), showing a trend toward inferior survival in Cluster 2 that did not reach statistical significance (log-rank *p* = 0.16)

In VenAza-treated patients, Cluster 2 demonstrated shorter EFS compared with Cluster 1, with median EFS of 6.87 months (95% CI 3.22–11.0) and 13.6 months (95% CI 1.97–not reached), respectively (log-rank *p* = 0.031) **(**Fig. 6A**)**. OS was also shorter in Cluster 2, with a median OS of 7.99 months (95% CI 3.42–20.2) compared with 23.6 months (95% CI 2.63–not reached) in Cluster 1; however, this difference did not reach significance (log-rank *p* = 0.16), indicating a trend toward inferior survival in Cluster 2 **(**Fig. 6B**)**.

Moreover, a separate analysis of individual genes within the VenAza-treated subgroup revealed higher *BIK* expression in non-responders (NR) compared with patients achieving complete remission (CR) or partial remission (PR) (Welch’s t-test, *p* = 0.0069) (Supplementary Table 9).

## D. Prognostic significance of individual apoptosis genes

**Fig. 7:**
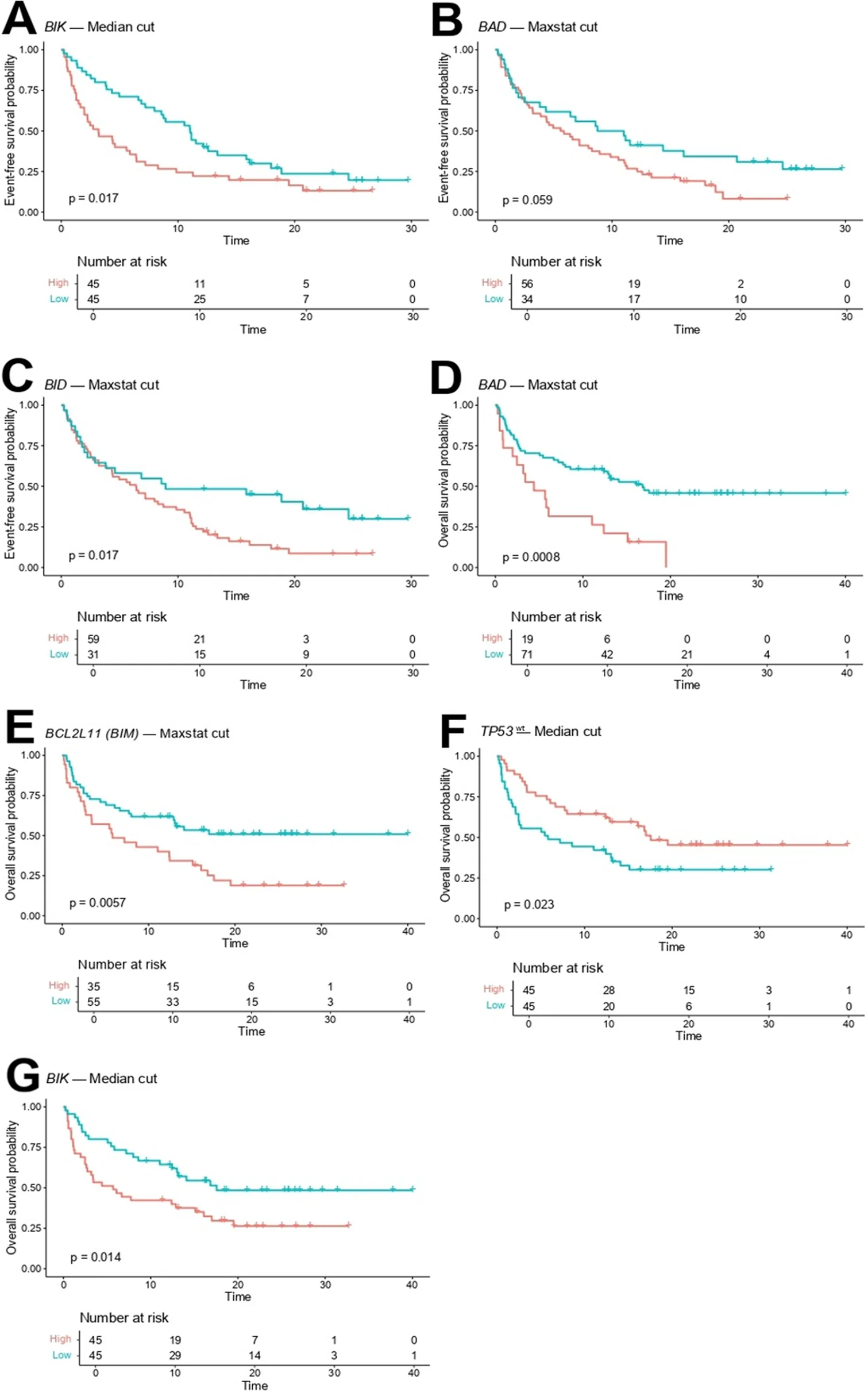
Kaplan–Meier curves showing event-free survival (EFS) and overall survival (OS) stratified by gene expression levels significant in univariate analyses. Patients were dichotomized into high- and low-expression groups using either median or maximally selected rank statistics (MaxStat) cut-offs, as indicated in each panel. (**A**) EFS according to *BIK* expression. (**B**) EFS according to *BAD* expression. (**C**) EFS according to *BID* expression. (**D**) OS according to *BAD* expression. (**E**) OS according to *BCL2L11* (*BIM*) expression. (**F**) OS according to *TP53*^wt^ expression. (**G**) OS according to *BIK* expression

In univariate Cox regression for EFS, higher expression of several pro-apoptotic genes was associated with inferior outcomes (Supplementary Table 10). Specifically, increased expression of *BIK* (HR 1.15, 95% CI 1.02–1.30, *p* = 0.0263; Fig. 7A), *BAD* (HR 1.38, 95% CI 1.06–1.81, *p* = 0.0168; Fig. 7B), and *BID* (HR 1.36, 95% CI 1.00–1.85, *p* = 0.0475; Fig. 7C) were significantly associated with shorter EFS. For OS, elevated expression of *BAD* (HR 1.41, 95% CI 1.05–1.89, *p* = 0.0210; Fig. 7D), *BCL2L11 (BIM)* (HR 1.30, 95% CI 1.04–1.62, *p* = 0.0229; Fig. 7E), and *BIK* (HR 1.19, 95% CI 1.03–1.38, *p* = 0.0162; Fig. 7G) was associated with worse survival, whereas higher *TP53*^wt^ expression was associated with improved OS (HR 0.72, 95% CI 0.53–0.97, *p* = 0.0324; Fig. 7F) (Supplementary Table 11).

In the multivariable model for EFS adjusted for clinical covariates, *BIK* expression remained independently associated with inferior outcome (HR 1.19, 95% CI 1.01–1.41, *p* = 0.0411) (Supplementary Table 12). Similarly, in the OS model, higher *BIK* expression was independently associated with shorter survival (HR 1.51, 95% CI 1.24–1.84, *p* = 0.0001) (Supplementary Table 13).

## Discussion

In this study, we prospectively evaluated mRNA expression of 15 apoptosis-related genes and their prognostic and therapeutic relevance in patients with newly diagnosed AML. Our findings highlight the complex regulation of apoptosis in AML and suggest that the clinical significance of apoptosis-related gene expression remains incompletely understood. The mutation rates in genes encoding apoptotic proteins are relatively low; therefore, dysregulation of this pathway is thought to arise primarily from altered gene expression, which prompted us to focus on transcriptional analysis [9, 10].

Using this approach, we identified two biologically and clinically distinct clusters of AML patients based on coordinated apoptosis-related gene expression patterns. These clusters differed not only in apoptosis-related gene expression profiles but also in prognosis and leukemic cell biology. Cluster 2 showed coordinated upregulation of multiple pro-apoptotic genes, including *BIM*,* PUMA*,* BID*,* BAX*,* BAD*, and *BIK*, together with increased expression of anti-apoptotic *MCL1* and *BCL-xL*, accompanied by reduced expression of executioner caspases (*CASP3*,* CASP7*) and lower expression of *BCL2*. The co-occurrence of these transcriptional abnormalities was associated with significantly inferior OS compared with Cluster 1, which displayed the opposite apoptosis-related transcriptional profile.

Reduced expression of *CASP3* and *CASP7* in Cluster 2 is consistent with observations in other malignancies, where loss or functional inhibition of executioner caspases is associated with defective apoptotic execution, treatment resistance, and poor prognosis [11]. In our cohort, elevated expression of pro-apoptotic genes in Cluster 2 did not translate into effective cell death, suggesting that insufficient caspase expression may limit completion of apoptosis. Both experimental and clinical studies identify impaired caspase activation as a key mechanism of apoptosis failure in cancer [12–15]. Although caspase activation was historically considered a “point of no return,” accumulating evidence indicates that insufficient activity may prevent completion of apoptosis despite the presence of upstream signals [16]. Leukemic cells lacking caspase-3 and − 7 display profound resistance to intrinsic and extrinsic apoptotic stimuli. Beyond their role as terminal effectors, caspases act as signal amplifiers required for full mitochondrial depolarization and efficient cell death [12], which may explain the discordance between transcriptional evidence of apoptotic priming and inferior clinical outcomes in our cohort. Experimental studies have also shown that prolonged exposure to chemotherapeutic agents may suppress caspase-3/7–dependent apoptosis and reduce pro-apoptotic p53 signaling, contributing to treatment resistance [13]. Restoration of caspase activity has been shown to re-sensitize malignant cells to apoptosis in experimental models, emphasizing the potential role of this pathway in treatment response [15]. Nevertheless, our findings are based on gene expression data and do not provide direct evidence of impaired apoptotic function. Therefore, the proposed mechanism should be considered a hypothesis requiring confirmation in functional studies.

Cluster 2 was characterized by increased expression of several pro-apoptotic genes, a lower frequency of *FLT3* mutations, and a lower leukemic blast burden, yet these patients had inferior overall survival and shorter event-free survival after VenAza treatment. Although lower *FLT3* mutation frequency and lower blast burden are generally considered favorable disease features, these characteristics did not translate into improved clinical outcomes in our cohort.

One possible explanation is that activation of upstream pro-apoptotic pathways does not necessarily result in effective apoptosis when CASP3 and CASP7 expression is reduced and alternative anti-apoptotic regulators are upregulated.

In our cohort, poorer survival in Cluster 2 was accompanied by increased expression of *MCL1* and *BCL2L1* together with reduced *BCL2* expression. This transcriptional profile is consistent with a shift in anti-apoptotic dependence that may also contribute to reduced sensitivity to BCL-2 inhibition. We therefore examined whether the baseline expression profile was associated with response to treatment with the BCL-2 inhibitor venetoclax. Patients treated with VenAza in Cluster 2 had significantly shorter EFS compared with Cluster 1. Because venetoclax selectively targets BCL-2, lower baseline *BCL2* expression, together with increased *MCL1* and *BCL2L1* expression, may have contributed to reduced therapeutic efficacy in this subgroup. Leukemic cells can bypass BCL-2 inhibition by shifting survival dependence toward alternative anti-apoptotic proteins, such as BCL-xL and MCL-1, thereby reducing response to venetoclax and impairing apoptotic execution [17]. This observation is consistent with the MAC-Score proposed by Waclawiczek et al., which integrates the relative expression of BCL-2, BCL-xL, and MCL-1 to estimate apoptotic dependency and treatment sensitivity. The score predicts initial response with a positive predictive value exceeding 97% and is associated with improved EFS in patients treated with a BCL2 inhibitor. In line with this model, higher BCL-2 dependence predicts better therapeutic response, whereas dominance of MCL-1 or BCL-xL reflects reduced mitochondrial priming and inferior outcomes [18].

Although our analysis relied on gene expression rather than protein-level measurements and was not restricted to leukemic stem cells, the transcriptional pattern observed in Cluster 2 reflects the principle underlying MAC scoring—namely, that AML prognosis may depend on the relative balance of anti-apoptotic regulators rather than the absolute expression of a single survival factor. Our findings support further investigation of MAC-based approaches and the development of prognostic indicators capturing apoptosis-related regulation beyond the LSC compartment.

However, because our analyses were limited to mRNA expression, confirmation at the protein level and in functional assays, such as BH3 profiling, will be valuable to establish whether the transcriptional profile observed in Cluster 2 reflects altered apoptotic priming.

This is particularly relevant in the clinical setting, as approximately one-third of treatment-naïve AML patients are refractory to VenAza [19]. Updated follow-up results from the VIALE-A trial additionally report a median duration of remission of approximately 18 months, with relapse occurring in most patients thereafter [20].

The immunophenotypic profile of Cluster 2 further suggests a shift toward a more differentiated, monocytic-like leukemic state. This subgroup showed higher expression of monocytic and mature myeloid markers, including CD11b, CD14, CD15, and CD64, together with reduced CD117 expression. Previous studies demonstrate that monocytic AML exhibits distinct mitochondrial survival regulation and reduced sensitivity to BCL-2–targeted therapy, partly due to increased reliance on alternative anti-apoptotic proteins MCL-1 and BCL-xL, and is associated with inferior OS [21, 22]. Consistent with these observations, Cluster 2 showed increased *MCL1* and *BCL2L1* expression together with reduced *BCL2*, suggesting a shift in anti-apoptotic dependence associated with monocytic differentiation. Patients in this cluster also had inferior clinical outcomes.

In Cluster 2, these features coexisted with a significantly lower frequency of *FLT3* mutations and a reduced leukemic burden at diagnosis—characteristics traditionally considered favorable. The observation that these patients nevertheless experienced inferior survival suggests that resistance to cell death, rather than proliferative capacity or mutational risk alone, may be a key determinant of outcome in this subgroup. The combined presence of monocytic differentiation, redistribution of anti-apoptotic signaling, and the observed apoptosis-related transcriptional profile therefore defines a biologically distinct AML subgroup in which differentiation state and apoptosis regulation interact to influence clinical outcome beyond conventional risk markers.

Beyond these broader apoptotic patterns, the prognostic significance of individual apoptosis-related genes in AML remains insufficiently characterized. In our cohort, higher *BIK* mRNA expression was independently associated with shorter OS and EFS in multivariable Cox regression models. Although *BIK* has traditionally been classified as a pro-apoptotic gene, accumulating evidence suggests that its overexpression may paradoxically correlate with poor clinical outcomes and tumor progression [23]. Similar associations have been reported in several malignancies, in which elevated *BIK* expression was linked to worse prognosis and chemoresistance in hematologic cancers and solid tumors [10, 23–26].

This apparent paradox may be related to the ability of BIK to induce incomplete or sublethal apoptosis rather than effective cell death. Pandya et al. demonstrated that BIK can trigger partial mitochondrial permeabilization and limited caspase activation, causing DNA damage without inducing effective apoptosis and promoting aggressive tumor phenotypes. Surviving cells may subsequently acquire genomic instability and stem cell–like features, leading to clonal expansion [24]. In addition, BIK-induced apoptotic stress may be mitigated by anti-apoptotic signaling or cytoprotective autophagy, enabling tumor cells to survive apoptotic pressure [27, 28]. Li et al. showed that pharmacologic inhibition of autophagy with agents such as hydroxychloroquine enhances the activity of venetoclax [29].

Consistent with these observations, we examined the association between *BIK* expression and treatment response in patients treated with VenAza. *BIK* expression was significantly higher in non-responders. This finding may relate to the functional classification of the BCL-2 family. BH3-only proteins can be divided into direct activators (e.g., BIM, BID, PUMA), which directly activate BAX and BAK, and sensitizers (e.g., BIK, BAD, NOXA), which promote apoptosis indirectly by binding anti-apoptotic proteins such as BCL-2, BCL-xL, and MCL-1 and releasing bound activators [30]. Because sensitizers such as BIK cannot directly activate BAX or BAK, high BIK expression alone may be insufficient to induce effective apoptosis, particularly during BCL-2 inhibition with venetoclax. Elevated BIK levels may also compete for binding sites required for the release of activator proteins such as PUMA and BIM, potentially limiting BAX/BAK activation and apoptotic response. However, direct experimental evidence supporting this mechanism in AML remains limited [31, 32]. Our findings are based on mRNA expression and demonstrate an association with clinical outcome rather than a direct effect of *BIK* on apoptotic regulation. Future studies incorporating functional models of BIK modulation and assessment of apoptotic activity will be important to clarify its role in AML.

Furthermore, our analysis indicated a trend toward improved OS associated with increased *TP53*^wt^ expression among individual apoptosis-related genes. This observation was not confirmed in multivariate Cox regression and therefore requires validation in larger AML cohorts. Although TP53 is a central regulator of apoptosis and the DNA damage response, wild-type expression may contribute to increased chemosensitivity and improved long-term outcomes [13, 33]. In clinical practice, therapeutic decisions in hematooncology frequently depend on *TP53* mutation status, whereas assessment of *TP53* mRNA expression has not yet found a clear prognostic application. Reduced *TP53* expression has been reported in nearly 90% of AML patients, suggesting that transcriptional downregulation may also contribute to AML pathogenesis [34, 35]. However, results remain inconsistent. Weng et al. reported that in 146 children with ALL, patients with *TP53* mRNA expression above the median had worse CR and OS compared with those with lower expression (85 vs. 96%, *p* = 0.025; 73 vs. 88%, *p* = 0.018), although mutation status was unknown [36]. Increased *TP53*^wt^ expression has also been associated with poorer prognosis in some gastrointestinal cancers, including gastric cancer [37]. The prognostic role of *TP53*^wt^ expression, therefore, remains unresolved and requires further investigation.

## Conclusion

Our findings suggest that apoptosis-related transcriptional patterns may have clinical relevance in AML and identify biologically distinct patient subgroups associated with different clinical outcomes. The association between higher *BIK* mRNA expression and poorer survival suggests that the role of apoptosis-related genes may be more complex than expected. Further validation at the protein level and in functional studies will be important to determine the biological and clinical significance of these findings.

## Supplementary Information

Below is the link to the electronic supplementary material.

Supplementary Material 1

Supplementary Material 2

Supplementary Material 3

## Data Availability

The datasets generated and analyzed during the current study are available from the corresponding author upon reasonable request. Additional methodological details are provided in the Supplementary Methods.
