# Supplementary material for: Apoptosis gene expression analysis identifies biologically distinct AML clusters and BIK as an independent prognostic marker
Source: Ann Hematol. 2026 Sep 10;105(11):452. doi: 10.1007/s00277-026-07268-7 (PMC13658304; doi:10.1007/s00277-026-07268-7)
Supplement: Supplementary file 1 [file 277_2026_7268_MOESM1_ESM.docx]

**Supplementary File 2**

**Supplementary Tables**

**Supplementary Table 1.** **Univariate Cox regression analysis of clinical variables associated with event-free survival (EFS).**

| **Variable** | **Comparison** | **HR (95% CI)** | **p-value** |
| --- | --- | --- | --- |
| Age >65 years | yes vs no | 1.57 (0.981–2.501) | 0.0604 |
| BM blasts at diagnosis | per 1% | 1.00 (0.995–1.013) | 0.3986 |
| ECOG ≥2 | yes vs 0–1 | 4.48 (2.737–7.332) | <0.0001 |
| Cytogenetic-molecular risk according to ELN 2022 | intermediate vs favorable | 2.00 (0.815–4.896) | 0.1303 |
|  | adverse vs favorable | 2.18 (0.916–5.166) | 0.0781 |
| *FLT3* mutation | present vs absent | 0.73 (0.379–1.399) | 0.3408 |
| HCT-CI | 2–3 vs 0–1 | 1.05 (0.630–1.744) | 0.8577 |
|  | ≥4 vs 0–1 | 1.26 (0.640–2.491) | 0.5008 |
| MDS-related cytogenetics | present vs absent | 1.40 (0.866–2.278) | 0.1683 |
| sAML | yes vs no | 1.18 (0.703–1.981) | 0.5311 |
| Sex | female vs male | 0.78 (0.488–1.254) | 0.3079 |
| Treatment | VenAza vs intensive | 1.61 (0.890–2.909) | 0.1151 |
|  | non-intensive vs intensive | 2.55 (1.448–4.508) | 0.0012 |

**Abbreviations:** HR, hazard ratio; CI, confidence interval; BM, bone marrow; ECOG, Eastern Cooperative Oncology Group performance status; ELN, European LeukemiaNet; HCT-CI, Hematopoietic Cell Transplantation–Comorbidity Index; FLT3, Fms-like tyrosine kinase 3; MDS, myelodysplastic syndrome; sAML, secondary Acute Myeloid Leukemia; VenAza, venetoclax + azacitidine.

**Supplementary Table 2.** **Univariate Cox regression analysis of clinical variables associated with overall survival (OS).**

| **Variable** | **Comparison** | **HR (95% CI)** | **p-value** |
| --- | --- | --- | --- |
| Age >65 years | yes vs no | 2.50 (1.416–4.415) | 0.0016 |
| PB blasts at diagnosis | per 1% | 1.00 (0.992–1.008) | 0.9980 |
| BM blasts at diagnosis | per 1% | 1.00 (0.993–1.014) | 0.5303 |
| ECOG ≥2 | yes vs 0–1 | 4.49 (2.595–7.779) | <0.0001 |
| Cytogenetic-molecular risk according to ELN 2022 | intermediate vs favorable | 2.64 (0.782–8.886) | 0.1181 |
|  | adverse vs favorable | 2.69 (0.816–8.855) | 0.1042 |
| *FLT3* mutation | present vs absent | 0.95 (0.441–2.061) | 0.9038 |
| HCT-CI | 2–3 vs 0–1 | 1.32 (0.740–2.365) | 0.3444 |
|  | ≥4 vs 0–1 | 1.31 (0.591–2.911) | 0.5040 |
| MDS-related cytogenetics | present vs absent | 1.99 (1.147–3.451) | 0.0144 |
| sAML | yes vs no | 1.56 (0.884–2.756) | 0.1246 |
| Sex | female vs male | 0.75 (0.435–1.305) | 0.3123 |
| Treatment | VenAza vs intensive | 2.58 (1.204–5.509) | 0.0147 |
|  | non-intensive vs intensive | 4.76 (2.330–9.709) | <0.0001 |

**Abbreviations:** HR, hazard ratio; CI, confidence interval; BM, bone marrow; ECOG, Eastern Cooperative Oncology Group performance status; ELN, European LeukemiaNet; HCT-CI, Hematopoietic Cell Transplantation–Comorbidity Index; FLT3, Fms-like tyrosine kinase 3; MDS, myelodysplastic syndrome; sAML, secondary Acute Myeloid Leukemia; VenAza, venetoclax + azacitidine.

**Supplementary Table 3.** **Differential expression of apoptosis-related genes between Cluster 1 and Cluster 2 identified by hierarchical clustering. Mean normalized gene expression values, fold change (FC), log2 fold change (log2FC), and statistical comparisons are shown.**

| **Gene** | **mean Cluster 1** | **SD Cluster 1** | **mean Cluster 2** | **SD Cluster 2** | **log2FC** | **FC**** | **p- value** | **FDR** |
| --- | --- | --- | --- | --- | --- | --- | --- | --- |
| *BAD* | -7.79 | 0.65 | -6.74 | 0.79 | 1.04 | 2.06 | 0.000 | 0.000 |
| *BID* | -3.35 | 0.49 | -2.50 | 0.72 | 0.85 | 1.80 | 0.000 | 0.000 |
| *BCL2L11 (BIM)* | -4.64 | 1.50 | -3.29 | 0.95 | 1.36 | 2.56 | 0.000 | 0.000 |
| *CASP3* | -1.71 | 0.95 | -2.66 | 0.86 | -0.95 | 0.52 | 0.000 | 0.000 |
| *BCL2* | -2.30 | 0.95 | -3.20 | 0.88 | -0.90 | 0.54 | 0.000 | 0.000 |
| *BBC3 (PUMA)* | -6.84 | 1.22 | -5.85 | 1.07 | 0.99 | 1.99 | 0.000 | 0.000 |
| *BIK* | -6.51 | 1.92 | -5.23 | 1.67 | 1.28 | 2.43 | 0.001 | 0.003 |
| *BAX* | -1.75 | 0.45 | -1.40 | 0.61 | 0.35 | 1.28 | 0.002 | 0.004 |
| *BCL2L1*  *(BCL-xL)* | -0.23 | 1.23 | 0.55 | 1.32 | 0.78 | 1.72 | 0.005 | 0.008 |
| *MCL1* | 0.15 | 0.95 | 0.64 | 1.02 | 0.49 | 1.40 | 0.021 | 0.030 |
| *CASP7* | -4.51 | 0.52 | -4.76 | 0.53 | -0.26 | 0.84 | 0.022 | 0.030 |
| *DIABLO* | -2.06 | 0.36 | -1.88 | 0.47 | 0.18 | 1.13 | 0.044 | 0.055 |
| *PMAIP1 (NOXA)* | -3.57 | 0.88 | -3.77 | 0.84 | -0.20 | 0.87 | 0.276 | 0.319 |
| *TP53*^wt^ | -0.94 | 0.63 | -0.83 | 0.89 | 0.11 | 1.08 | 0.495 | 0.530 |
| *BAK1* | -8.06 | 4.45 | -8.01 | 3.71 | 0.05 | 1.04 | 0.955 | 0.955 |

**Abbreviations:** SD, standard deviation; log2FC, log2 fold change; FC, fold change; FDR, false discovery rate. **Fold change (FC) was calculated as the ratio of mean gene expression in Cluster 2 to that in Cluster 1 (FC = Cluster 2 / Cluster 1). Log2 fold change (log2FC) was calculated as log2(FC). Thus, FC > 1 indicates higher expression in Cluster 2, whereas FC < 1 indicates lower expression relative to Cluster 1.

**Supplementary Table 4.** **Comparison of mutational landscape between apoptosis gene expression clusters.**

| **Gene** | **N mut C1** | **freq C1** | **N mut C2** | **freq C2** | **p-value** |
| --- | --- | --- | --- | --- | --- |
| *NPM1* | 9 | 0.36 | 8 | 0.333 | 1.0000 |
| *FLT3(ITD+TKD)* | 13 | 0.52 | 3 | 0.125 | 0.0050 |
| *FLT3-ITD* | 12 | 0.48 | 3 | 0.125 | 0.0120 |
| *FLT3-TKD* | 2 | 0.08 | 0 | 0 | 0.4900 |
| *IDH1/2* | 11 | 0.44 | 6 | 0.25 | 0.2320 |
| *IDH1* | 5 | 0.2 | 3 | 0.125 | 0.7020 |
| *IDH2* | 7 | 0.28 | 3 | 0.125 | 0.2890 |
| bZip in frame *CEBPA* | 2 | 0.08 | 0 | 0 | 0.4900 |
| non-bZip *CEBPA* | 3 | 0.12 | 1 | 0.042 | 0.6090 |
| frameshift *CEBPA* | 5 | 0.2 | 1 | 0.042 | 0.1890 |
| *JAK2* | 4 | 0.16 | 4 | 0.167 | 1.0000 |
| *CALR* | 0 | 0 | 1 | 0.042 | 0.4900 |
| *DNMT3A* | 6 | 0.24 | 4 | 0.167 | 0.7250 |
| *GATA2* | 1 | 0.04 | 0 | 0 | 1.0000 |
| *KIT* | 1 | 0.04 | 1 | 0.042 | 1.0000 |
| *CSF3R* | 1 | 0.04 | 0 | 0 | 1.0000 |
| *WT1* | 1 | 0.04 | 2 | 0.083 | 0.6090 |
| *MPL* | 0 | 0 | 1 | 0.042 | 0.4900 |
| *NRAS* | 2 | 0.08 | 6 | 0.25 | 0.1380 |
| *KRAS* | 2 | 0.08 | 3 | 0.125 | 0.6670 |
| *HRAS* | 0 | 0 | 0 | 0 | NA |
| *PHF6* | 4 | 0.16 | 6 | 0.25 | 0.4960 |
| *PRPF8* | 0 | 0 | 2 | 0.083 | 0.2350 |
| *PTPN11* | 1 | 0.04 | 2 | 0.083 | 0.6090 |
| *BRAF* | 1 | 0.04 | 2 | 0.083 | 0.6090 |
| *SETBP1* | 0 | 0 | 0 | 0 | NA |
| *CBL* | 1 | 0.04 | 0 | 0 | 1.0000 |
| *NF1* | 1 | 0.04 | 1 | 0.042 | 1.0000 |
| *ETV6* | 4 | 0.16 | 3 | 0.125 | 1.0000 |
| *IKZF1* | 1 | 0.04 | 1 | 0.042 | 1.0000 |
| *TET2* | 5 | 0.2 | 5 | 0.208 | 1.0000 |
| *RB1* | 1 | 0.04 | 0 | 0 | 1.0000 |
| *ASXL1* | 1 | 0.04 | 2 | 0.083 | 0.6090 |
| *BCOR* | 4 | 0.16 | 4 | 0.167 | 1.0000 |
| *EZH2* | 7 | 0.28 | 2 | 0.083 | 0.1380 |
| *RUNX1* | 8 | 0.32 | 4 | 0.167 | 0.3210 |
| *SF3B1* | 0 | 0 | 3 | 0.125 | 0.1100 |
| *SRSF2* | 5 | 0.2 | 3 | 0.125 | 0.7020 |
| *STAG2* | 4 | 0.16 | 4 | 0.167 | 1.0000 |
| *U2AF1* | 3 | 0.12 | 1 | 0.042 | 0.6090 |
| *ZRSR2* | 3 | 0.12 | 2 | 0.083 | 1.0000 |

**Abbreviations:** N mut, number of mutated cases; freq, mutation frequency; C1, Cluster 1; C2, Cluster 2; ITD, internal tandem duplication; TKD, tyrosine kinase domain; bZip, basic leucine zipper domain; NA, not applicable.

**Supplementary Table 5. Comparison of immunophenotypic profiles between apoptosis gene expression clusters.**

| **Marker** | **Me**  **Cluster 1** | **Q1**  **Cluster 1** | **Q3**  **Cluster 1** | **Me**  **Cluster 2** | **Q1**  **Cluster 2** | **Q3**  **Cluster 2** | **p-value** | **FDR** |
| --- | --- | --- | --- | --- | --- | --- | --- | --- |
| CD11b | 2.5 | 0.5 | 4.5 | 9.4 | 4.3 | 36.5 | 0.0001 | 0.0017 |
| CD15 | 6.8 | 2.0 | 18.2 | 23.5 | 11.3 | 40.9 | 0.0004 | 0.0036 |
| CD2 | 0.2 | 0.1 | 0.6 | 0.9 | 0.2 | 6.5 | 0.0019 | 0.0091 |
| CD14 | 0.2 | 0.1 | 1.1 | 1.1 | 0.3 | 12.1 | 0.0022 | 0.0091 |
| CD64 | 9.4 | 2.2 | 19.6 | 24.6 | 8.9 | 77.6 | 0.0024 | 0.0091 |
| CD117 | 85.2 | 69.0 | 91.9 | 72.0 | 25.5 | 83.3 | 0.0060 | 0.0190 |
| CD11c | 17.8 | 4.6 | 46.7 | 34.2 | 11.8 | 74.8 | 0.0291 | 0.0790 |
| CD34 | 73.4 | 17.7 | 90.0 | 34.0 | 1.0 | 79.3 | 0.0394 | 0.0937 |
| CD34/CD117 | 63.5 | 5.7 | 83.6 | 15.5 | 2.6 | 70.2 | 0.0703 | 0.1485 |
| CD56 | 0.4 | 0.1 | 4.2 | 0.9 | 0.2 | 17.3 | 0.1586 | 0.2990 |
| HLA-DR | 72.8 | 61.3 | 85.0 | 80.9 | 52.5 | 91.0 | 0.1771 | 0.2990 |
| CD123 | 43.0 | 9.3 | 87.8 | 26.0 | 9.4 | 69.9 | 0.1888 | 0.2990 |
| CD31 | 97.9 | 89.5 | 98.7 | 98.2 | 94.4 | 99.2 | 0.2916 | 0.4262 |
| CD13 | 90.5 | 79.8 | 95.0 | 86.3 | 70.9 | 95.0 | 0.3909 | 0.5083 |
| CD22 | 0.3 | 0.1 | 1.3 | 0.5 | 0.2 | 1.3 | 0.4013 | 0.5083 |
| CD7 | 0.5 | 0.1 | 36.1 | 0.5 | 0.1 | 1.9 | 0.5308 | 0.6054 |
| CD19 | 0.8 | 0.2 | 4.6 | 0.7 | 0.3 | 1.4 | 0.5417 | 0.6054 |
| CD38 | 93.8 | 84.6 | 96.5 | 94.9 | 87.0 | 97.4 | 0.5930 | 0.6259 |
| CD33 | 90.9 | 50.8 | 98.8 | 96.6 | 76.6 | 98.6 | 0.6484 | 0.6484 |

**Abbreviations:** Me, median; Q1, first quartile; Q3, third quartile; FDR, false discovery rate.

**Supplementary Table 6.** **Baseline clinical characteristics of patients according to apoptosis gene expression cluster assignment.**

| **Variable** | **Cluster 1 (N=41)** | **Cluster 2 (N=49)** | **p-value** |
| --- | --- | --- | --- |
| Sex, female - n (%) | 17 (41.5) | 22 (44.9) | 0.9090 |
| Age at diagnosis - median (IQR) | 63.0 (55.0–68.0) | 68.0 (59.0–77.0) | 0.0792 |
| Age >65 years - n (%) | 20 (48.8) | 32 (65.3) | 0.1720 |
| ECOG - n (%) | 1.0 (1.0–2.0) | 1.0 (1.0–2.0) | 0.8920 |
| HCT-CI - n (%) | 1.0 (0.0–2.0) | 2.0 (0.0–3.0) | 0.1360 |
| alloHSCT - n (%) | 15 (36.6) | 11 (22.4) | 0.2150 |
| Response (CR vs PR/NR/ED) | 21 (51.2) | 21 (42.9) | 0.5620 |
| sAML - n (%) | 8 (19.5) | 17 (34.7) | 0.1720 |
| MDS-related cytogenetics - n (%) | 14 (35.0) | 20 (42.6) | 0.6180 |
| Cytogenetic-molecular risk according to ELN 2022 - n (%)  favorable risk intermediate risk adverse risk | 5 (12.5)  16 (40.0)  19 (47.5) | 6 (12.8)  16 (34.0)  25 (53.2) | 0.9100 |
| Laboratory parameters at diagnosis | Median (IQR) | |  |
| Blasts, PB (%) | 53.0 (6.0–79.0) | 9.0 (1.0–24.0) | 0.0004 |
| Blasts, BM (%) | 54.0 (31.0–76.0) | 37.0 (19.0–54.0) | 0.0039 |
| WBC (×10⁹/L) | 26.0 (4.0–84.0) | 9.0 (3.0–48.0) | 0.2010 |
| ANC (×10⁹/L) | 1.6 (0.4–4.8) | 2.8 (0.8–8.3) | 0.2290 |
| Hb (g/dL) | 9.0 (8.3–10.4) | 8.7 (7.6–9.5) | 0.1350 |
| Plt (×10⁹/L) | 48.0 (21.0–76.0) | 59.0 (35.0–114.0) | 0.1520 |
| CRP (mg/L) | 24.0 (7.5–78.0) | 17.0 (6.0–78.2) | 0.8050 |
| Elevated CRP - n (%) | 32 (78.0) | 37 (75.5) | 0.9730 |
| LDH (U/L) | 462.5 (248.0–858.8) | 391.0 (196.5–705.0) | 0.3070 |
| Elevated LDH - n (%) | 28 (68.3) | 32(65.3) | 0.9400 |
| Uric acid (mg/dL) | 5.3 (4.2–6.7) | 6.0 (5.1–7.2) | 0.0601 |
| Elevated uric acid - n (%) | 14 (34.1) | 24 (49.0) | 0.2280 |
| Albumin (g/dL) | 3.8 (3.4–4.2) | 3.8 (3.4–4.1) | 0.8150 |
| Low albumin - n (%) | 15 (37.5) | 16 (33.3) | 0.8550 |

**Abbreviations:** IQR, interquartile range; ECOG, Eastern Cooperative Oncology Group performance status; HCT-CI, Hematopoietic Cell Transplantation–Comorbidity Index; alloHSCT, allogeneic hematopoietic stem cell transplantation; CR, complete remission; PR, partial remission; NR, no response; ED, early death; sAML, secondary acute myeloid leukemia; MDS, myelodysplastic syndrome; ELN, European LeukemiaNet; PB, peripheral blood; BM, bone marrow; WBC, white blood cell count; ANC, absolute neutrophil count; Hb, hemoglobin; Plt, platelet count; CRP, C-reactive protein; LDH, lactate dehydrogenase.

**Supplementary Table 7.** **Multivariable Cox regression model for event-free survival including cluster assignment and clinical covariates.**

| **Variable** | **HR** | **95% CI** | **p-value** |
| --- | --- | --- | --- |
| Cluster 2 vs 1 | 2.10 | 1.26–3.49 | 0.0044 |
| Age >65 years | 0.88 | 0.51–1.54 | 0.6625 |
| ECOG ≥2 | 4.33 | 2.47–7.58 | <0.0001 |
| Cytogenetic-molecular risk according to ELN 2022  Favorable + Intermediate vs Adverse | 1.08 | 0.68–1.72 | 0.7331 |
| VenAza vs Intensive | 1.43 | 0.72–2.82 | 0.2979 |
| Non-intensive vs Intensive | 1.74 | 0.87–3.48 | 0.1160 |

**Abbreviations:** HR, hazard ratio; CI, confidence interval; ECOG, Eastern Cooperative Oncology Group performance status; ELN, European LeukemiaNet; VenAza, venetoclax + azacitidine.

**Supplementary Table 8.** **Multivariable Cox regression model for overall survival including cluster assignment and clinical covariates.**

| **Variable** | **HR** | **95% CI** | **p-value** |
| --- | --- | --- | --- |
| Cluster 2 vs 1 | 2.14 | 1.19–3.87 | 0.0113 |
| Age >65 years | 0.78 | 0.39-1.56 | 0.4857 |
| ECOG ≥2 | 4.00 | 2.14-7.51 | <0.0001 |
| Cytogenetic-molecular risk according to ELN 2022  Favorable + Intermediate vs Adverse | 0.86 | 0.50-1.47 | 0.5772 |
| VenAza vs Intensive | 1.75 | 0.78–3.97 | 0.1740 |
| Non-intensive vs Intensive | 2.25 | 0.94–5.37 | 0.0678 |
| MDS-related cytogenetics | 1.71 | 0.97–3.01 | 0.0652 |

**Abbreviations:** HR, hazard ratio; CI, confidence interval; ECOG, Eastern Cooperative Oncology Group performance status; ELN, European LeukemiaNet; VenAza, venetoclax + azacitidine; MDS, myelodysplastic syndrome.

**Supplementary Table 9. Comparison of normalized *BIK* expression levels according to treatment response among patients treated with azacitidine + venetoclax. Comparison performed using Welch’s t-test.**

| **Gene** | **CR/PR achieved** | | | **NR** | | | **t-stat** | **p-value** |
| --- | --- | --- | --- | --- | --- | --- | --- | --- |
|  | n (%) | Mean | SD | n (%) | Mean | SD |  |  |
| *BIK* | 18 (72.0%) | -6.546 | 1.564 | 7 (28.0%) | -5.079 | 0.842 | -3.011 | 0.0069 |

**Abbreviations:** CR, complete remission; PR, partial remission; NR, no response; SD, standard deviation.

**Supplementary Table 10.** **Univariate Cox regression analysis of apoptosis-related gene expression and event-free survival (EFS). Hazard ratios represent risk per unit increase in normalized gene expression.**

| **Gene** | **HR** | **95% CI** | **p-value** |
| --- | --- | --- | --- |
| *BAD* | 1.38 | 1.06–1.81 | 0.0168 |
| *BIK* | 1.15 | 1.02–1.30 | 0.0263 |
| *BID* | 1.36 | 1.00–1.85 | 0.0475 |
| *BIM (BCL2L11)* | 1.17 | 0.98–1.41 | 0.0864 |
| *TP53*^wt^ | 0.77 | 0.58–1.03 | 0.0788 |
| *BCL-xL (BCL2L1)* | 0.87 | 0.73–1.03 | 0.1072 |
| *MCL1* | 1.19 | 0.95–1.49 | 0.1284 |
| *DIABLO* | 1.51 | 0.85–2.70 | 0.1630 |
| *BAX* | 1.32 | 0.84–2.08 | 0.2223 |
| *CASP3* | 0.87 | 0.68–1.10 | 0.2467 |
| *BAK1* | 1.02 | 0.96–1.08 | 0.5068 |
| *PUMA (BBC3)* | 1.07 | 0.87–1.30 | 0.5265 |
| *CASP7* | 1.16 | 0.73–1.85 | 0.5178 |
| *BCL2* | 0.95 | 0.76–1.19 | 0.6499 |
| *NOXA (PMAIP1)* | 0.99 | 0.76–1.29 | 0.9258 |

**Abbreviations:** HR, hazard ratio; CI, confidence interval.

**Supplementary Table 11.** **Univariate Cox regression analysis of apoptosis-related gene expression and overall survival (OS).**

| **Gene** | **HR** | **95% CI** | **p-value** |
| --- | --- | --- | --- |
| *BAD* | 1.41 | 1.05–1.89 | 0.0210 |
| *BIM (BCL2L11)* | 1.30 | 1.04–1.62 | 0.0229 |
| *BIK* | 1.19 | 1.03–1.38 | 0.0162 |
| *TP53*^wt^ | 0.72 | 0.53–0.97 | 0.0324 |
| *BID* | 1.29 | 0.93–1.79 | 0.1318 |
| *MCL1* | 1.17 | 0.91–1.51 | 0.2297 |
| *BCL2* | 0.88 | 0.68–1.14 | 0.3237 |
| *BCL-xL (BCL2L1)* | 0.91 | 0.74–1.12 | 0.3739 |
| *CASP7* | 0.77 | 0.45–1.31 | 0.3411 |
| *BAK1* | 1.02 | 0.96–1.09 | 0.4593 |
| *DIABLO* | 1.25 | 0.64–2.43 | 0.5177 |
| *PUMA (BBC3)* | 1.06 | 0.85–1.33 | 0.6038 |
| *CASP3* | 0.93 | 0.70–1.24 | 0.6263 |
| *BAX* | 0.94 | 0.56–1.56 | 0.7965 |
| *NOXA (PMAIP1)* | 0.96 | 0.71–1.32 | 0.8199 |

**Abbreviations:** HR, hazard ratio; CI, confidence interval.

**Supplementary Table 12**. **Multivariable Cox regression model for event-free survival including clinical covariates and apoptosis gene expression.**

| **Variable** | **HR** | **95% CI** | **p-value** |
| --- | --- | --- | --- |
| Age >65 years | 1.17 | 0.65-2.10 | 0.6087 |
| ECOG ≥2 | 4.29 | 2.46-7.50 | <0.0001 |
| Cytogenetic-molecular risk according to ELN 2022  Favorable + Intermediate vs Adverse | 1.13 | 0.71–1.82 | 0.6027 |
| VenAza vs intensive | 1.54 | 0.75–3.17 | 0.2373 |
| Non-intensive vs intensive | 1.80 | 0.88–3.72 | 0.1092 |
| *BAD* expression | 1.12 | 0.76–1.62 | 0.5454 |
| *BIK* expression | 1.19 | 1.01–1.41 | 0.0411 |
| *BID* expression | 1.35 | 0.96–1.91 | 0.0843 |

**Abbreviations:** HR, hazard ratio; CI, confidence interval; ECOG, Eastern Cooperative Oncology Group performance status; ELN, European LeukemiaNet; VenAza, venetoclax + azacitidine.

**Supplementary Table 13.** **Multivariable Cox regression model for overall survival (OS) including clinical covariates and apoptosis gene expression.**

| **Variable** | **HR** | **95% CI** | **p-value** |
| --- | --- | --- | --- |
| Age >65 years | 0.85 | 0.41–1.78 | 0.6711 |
| ECOG ≥2 | 3.96 | 2.10–7.47 | <0.0001 |
| Cytogenetic-molecular risk according to ELN 2022  Favorable + Intermediate vs Adverse | 0.82 | 0.46–1.46 | 0.5048 |
| VenAza vs intensive | 2.03 | 0.85–4.86 | 0.1115 |
| Non-intensive vs intensive | 3.65 | 1.50–8.88 | 0.0044 |
| MDS-related cytogenetics | 2.06 | 1.13-3.77 | 0.0186 |
| *BAD* expression | 0.75 | 0.49–1.13 | 0.1685 |
| *BIM* expression | 1.07 | 0.85–1.35 | 0.5503 |
| *BIK* expression | 1.51 | 1.24–1.84 | 0.0001 |
| *TP53*^wt^ expression | 0.75 | 0.51–1.09 | 0.1263 |

**Abbreviations:** HR, hazard ratio; CI, confidence interval; ECOG, Eastern Cooperative Oncology Group performance status; ELN, European LeukemiaNet; VenAza, venetoclax + azacitidine; MDS, myelodysplastic syndrome.
