# Supplementary material for: Apoptosis gene expression analysis identifies biologically distinct AML clusters and BIK as an independent prognostic marker
Source: Ann Hematol. 2026 Sep 10;105(11):452. doi: 10.1007/s00277-026-07268-7 (PMC13658304; doi:10.1007/s00277-026-07268-7)
Supplement: Supplementary file 2 [file 277_2026_7268_MOESM2_ESM.docx]

Supplementary File 1 – Supplementary Methods

1. *Treatment regimens*

All patients received treatment according to the recommendations of the Polish Adult Leukemia Group (PALG) recommendations. Patients with *FLT3*-ITD or *FLT3*-TKD mutations received DA induction based on daunorubicin 60mg/m^2^ (days 1-3), and cytarabine (AraC) 200mg/m^2^ (days 1-7) in combination with midostaurin 50mg twice daily (days 8-21), while patients with core-binding factor (CBF) AML were treated with DA induction with one dose of gemtuzumab ozogamicin (GO) 5mg/m^2^ on day 1. The remaining 33 fit patients received DA induction in combination with cladribine 5mg/m^2^ (days 1-5) – DAC regimen. Based on treatment response, patients received, they continued with second induction or consolidation treatment based on intermediate-dose AraC (1-1.5g/m^2^, 6 doses). Twenty-five patients were treated with azacitidine 75mg/m^2^ (days 1-7) plus venetoclax 400mg (14 to 28 days depending on response and hematologic toxicity). The remaining 32 patients received non-intensive treatment, including azacitidine monotherapy, low doses of cytarabine (LDAC), or cytoreduction with best supportive care (BSC).

2. *Preparation of mononuclear cells and total RNA isolation*

Total RNA was isolated directly from approximately 10 million human bone marrow mononuclear cells (BMMCs). Bone marrow samples were collected in EDTA-containing vacutainers and diluted with PBS (10 ml bone marrow + 30 ml cold PBS). Fifteen milliliters of Histopaque was added to a 50 mL Falcon tube and overlaid with 25 mL of diluted bone marrow, then centrifuged at 800 × g for 30 minutes at room temperature without brake. The plasma layer was carefully removed, and the buffy coat containing mononuclear cells was transferred to a new 50 mL tube. An equal volume of PBS was added, and the sample was centrifuged at 400 × g for 10 minutes at 20°C. The cell pellet was resuspended in RNAprotect Cell Reagent (Qiagen, Germantown, USA) and stored at −80°C. Total RNA isolation was performed using the RNeasy Mini Kit (Qiagen) according to the manufacturer’s protocol. RNA was eluted in 50 µL of RNase-free water. RNA concentration was measured fluorimetrically with the Qubit 4.0 fluorometer. Total RNA was subsequently used for reverse transcription.

3. *Reverse transcription*

Reverse transcription was performed using the High-Capacity cDNA Reverse Transcription Kit (Applied Biosystems, Thermo Fisher Scientific, Waltham, MA, USA) according to the manufacturer’s protocol. The reaction volume was 20 µL per sample, with 2,000 ng of total RNA used as the sample input per 20 µL of the reverse transcription reaction. Reactions were performed on a QuantStudio 7 system (ThermoFisher Scientific). The resulting cDNA was stored at −20°C.

4. *Selection of reference genes*

Reference genes were selected from 32 candidate genes with the most stable expression in samples from six patients using the TaqMan™ Array Human Endogenous Control plate (ThermoFisher Scientific). NormiRazor software was used for statistical evaluation, employing three algorithms: geNorm, BestKeeper, and NormFinder [8]. The *MRPL19* and *PPIA* gene pair demonstrated the highest stability and was selected as the reference.

5. *Real-time PCR*

The mRNA expression levels of *BCL2L11 (BIM), BID, BIK, BBC3 (PUMA), PMAIP1 (NOXA), BAD, BAX, BAK1, BCL2, BCL2L1 (BCL-xL), MCL1, SMAC/DIABLO, CASP3, CASP7,* and *TP53*^wt^ genes were quantified and normalized to *MRPL19* and *PPIA*.

Real-time PCR reactions were performed using ready-to-use TaqMan® Gene Expression Assays (Applied Biosystems- Thermo Fisher Scientific, Waltham, MA, USA). The PCR mixture consisted of 10 µL of 2X TaqMan™ GeneExpression Master Mix (Applied Biosystems, Thermo Fisher Scientific, Waltham, MA, USA), 1 µL of the appropriate 20X TaqMan® Assay, and 1 µL of cDNA template. The PCR was performed on the QuantStudio™ 7 system (Applied Biosystems - Thermo Fisher Scientific). All measurements were performed in technical duplicates under the following conditions: an initial denaturation step at 95°C for 10 minutes, followed by 40 cycles of denaturation (95°C, 15 s), a single annealing and extension step (60°C for 1 minute). Fluorescence signal detection was performed after each cycle.

6. *Data analysis*

Data were normalized based on the mean Ct values of the two selected reference genes. Quantitative analysis was performed using threshold cycle (Ct) values obtained during the exponential amplification phase. The Ct value was defined as the cycle at which the fluorescence signal exceeded a fixed threshold. A Ct value ≤ 37 was considered a positive expression signal. Relative expression levels were calculated using the ΔCt method: ∆Ct = Ct(reference) – Ct(mRNA of interest). Fold changes (FC) were calculated using the ΔΔCt method.

7. *Statistical analysis*

Continuous variables were summarized using medians and interquartile ranges (IQR), whereas categorical variables were reported as counts and percentages. Between-group comparisons were performed using the Mann–Whitney U test for continuous variables and the Chi2 or Fisher’s exact test for categorical variables, as appropriate.

Event-free survival (EFS) was defined as the time from diagnosis to relapse, death, or last follow-up, and overall survival (OS) as the time from diagnosis to death from any cause or last follow-up. Survival curves were estimated with the Kaplan–Meier method and compared with the log-rank test. Kaplan–Meier curves were generated after dichotomizing gene expression at the median value. When median-based stratification did not yield significant separation, an optimal cut-off was determined using maximally selected rank statistics (maxstat method).

Univariate Cox proportional hazards regression models assessed associations between clinical variables and survival outcomes. Variables with clinical relevance and p < 0.1 in univariate analyses were included in multivariable Cox regression models. Hazard ratios (HRs) were reported with 95% confidence intervals (CIs).

Unsupervised hierarchical clustering of apoptosis-related gene expression was performed using Spearman distance and complete linkage. The resulting dendrogram was partitioned into two clusters corresponding to the first major split of the hierarchical tree. Differential gene expression between clusters was assessed using Welch’s t-test, with false discovery rate (FDR) correction for multiple testing. Comparisons of immunophenotypic marker expression between clusters were performed using the Mann–Whitney U test with adjustment for multiple comparisons. All statistical tests were two-sided, and a p-value < 0.05 was considered statistically significant.

All analyses were performed using R software (version 4.4.2; R Foundation for Statistical Computing, Vienna, Austria).
