# Supplementary material for: Apoptosis gene expression analysis identifies biologically distinct AML clusters and BIK as an independent prognostic marker
Source: Ann Hematol. 2026 Sep 10;105(11):452. doi: 10.1007/s00277-026-07268-7 (PMC13658304; doi:10.1007/s00277-026-07268-7)
Supplement: Supplementary file 3 [file 277_2026_7268_MOESM3_ESM.docx]

**Supplementary File 3**

**Supplementary Figures**

**Supplementary Figure 1. Differences in the expression of apoptosis-related genes between identified clusters shown as boxplots.** The Y-axis represents cycle threshold (Ct) values (higher Ct = higher expression). Points indicate individual samples, the line inside each box marks the median, and boxes show the interquartile range. *P* values were calculated using Welch’s t-test. (A) *BAD*, (B) *PUMA/BBC3*, (C) *MCL1*, (D) *CASP3*, (E) *BIM/BCL2L11*, (F) *BIK*, (G) *BID*, (H) *BCL-xL/BCL2L1*, (I) *BCL2*, (J) *BAX*, (K) *CASP7*.
